# Artificial Jaundice

**Published:** 1862-07

**Authors:** 


Artificial Jaundice.—Dr. Harley reminded the members, of the case of complete obstruction of the bile and gall ducts, occurring in a gentleman aged about fifty, which he had brought before the Society at the last meeting, and where crystals of leucine and tyrosine were not only present in the urine, but even in the liver itself. As Freriches had pointed out that these products are of important diagnostic value in hepatic affections, Dr. Harley was anxious to study their pathology more carefully; and being well aware that the physiologist has it in his power to produce almost any pathological state at pleasure, he tried to imitate in an animal the effects produced in man by the obstruction of the bile ducts. Artificial jaundice has been usually induced either by ligaturing the gall ducts or injecting bile into the circulation; but as both of these methods were in the present instance objectionable,—the first, on account of the constitutional disturbance induced by the severity of the operation; the second, from the bile being all at once thrown into the circulation, and producing toxic effects, besides its being too rapidly eliminated by the urine. Dr. Harley adopted another plan, which came much nearer to the state induced by disease in man. He took the bile of three healthy dogs, and injected it under the skin of a fourth. In this case the effects of the operation were almost *nil* and the bile was at the same time placed in a position favorable for its slow absorption just as in the human subjects. During the first two days the animal remained comparatively well, the urine was normal, and contained neither bile pigment nor biliary acids. On the third day, however, the animal became ill; and on the fourth. jaundice set in. He died on the fifth day. After death the urine was found to contain not only bile pigment and billiary acids, but also the diseased products leucine and tyrosine, and, what was more interesting still, the urine was found to be loaded with sugar, just as is occasionally met with in the human subject. The urine containing the crystals described was exhibited under the microscope.—*London Lancet.*

